# Phenotypic switching in *Candida tropicalis* alters host-pathogen interactions in a *Galleria mellonella* infection model

**DOI:** 10.1038/s41598-019-49080-6

**Published:** 2019-08-29

**Authors:** Hugo F. Perini, Alane T. P. Moralez, Ricardo S. C. Almeida, Luciano A. Panagio, Admilton O. G. Junior, Fernando Gomes Barcellos, Luciana Furlaneto-Maia, Marcia C. Furlaneto

**Affiliations:** 1 Department of Microbiology, Center of Biological Sciences, Londrina State University, C.P. 6001, 86051990, Londrina, Paraná Brazil; 2 Department of General Biology, Center of Biological Sciences, Londrina State University, C.P. 6001, 86051990, Londrina, Paraná Brazil; 3 Department of Food Technology, Technological Federal University of Paraná, 86036-370, Londrina, Paraná Brazil

**Keywords:** Fungal host response, Fungi

## Abstract

*Candida tropicalis* is a human pathogen associated with high mortality rates. We have reported a switching system in *C*. *tropicalis* consisting of five morphotypes – the parental, switch variant (crepe and rough), and revertant (crepe and rough) strains, which exhibited altered virulence in a *Galleria mellonella* model. Here, we evaluate whether switching events may alter host-pathogen interactions by comparing the attributes of the innate responses to the various states. All switched strains induced higher melanization in *G*. *mellonella* larvae than that induced by the parental strain. The galiomicin expression was higher in the larvae infected with the crepe and rough morphotypes than that in the larvae infected with the parental strain. Hemocytes preferentially phagocytosed crepe variant cells over parental cells *in vitro*. In contrast, the rough variant cells were less phagocytosed than the parental strain. The hemocyte density was decreased in the larvae infected with the crepe variant compared to that in the larvae infected with the parental strain. Interestingly, larvae infected with the revertant of crepe restored the hemocyte density levels that to those observed for larvae infected with the parental strain. Most of the switched strains were more resistant to hemocyte candidacidal activity than the parental strain. These results indicate that the switch states exhibit similarities as well as important differences during infection in a *G*. *mellonella* model.

## Introduction

Phenotypic switching is a strategy that allows microorganisms to undergo adaptation to environmental changes. In fungi, this event is random, reversible and defined as the emergence of colonies with altered morphology at rates higher than the somatic mutation rates^1^. Currently, the best characterized fungal phenotypic switch is the ‘white-opaque’ transition of the opportunistic pathogen *Candida albicans*^2^, where pleiotropic effects on virulence have been described^3,4^.

*Candida tropicalis* is a clinically relevant species representing the second or third etiological agent of candidemia, especially in tropical regions^5–7^. *C*. *tropicalis* is genetically similar to *C*. *albicans* and can also undergo an epigenetic switch between white and opaque phenotypic states^8–10^. Cells from the white parental colonies appear round and have a smooth cell surface, whereas cells from the opaque (darker-colored) colonies are elongated and exhibit a spotted cell surface^8^. The white-opaque switch in *C*. *tropicalis* regulates a cryptic program of sexual reproduction, where only cells in the opaque state undergo efficient mating^8^. The white-opaque transition is also associated with sexual biofilm formation in *C*. *tropicalis*, in which biofilms are formed exclusively by opaque cells^10^. In addition, similar to *C*. *albicans*, *C*. *tropicalis* makes use of a tristable switching system between the white, gray, and opaque cell types^11^. *C*. *tropicalis* gray cells exhibit an intermediate level of mating capability and virulence in a mouse infection model compared to that of white and opaque cell types^11^.

In addition to the white-opaque and white-gray-opaque phenotypic transitions, *C*. *tropicalis* can undergo multiple forms of phenotypic switching. Soll *et al*.^12^ demonstrated for the first time that *C*. *tropicalis* can undergo a high-frequency switch during the course of a prolonged *Candida* infection in an immunocompromised host. Our group has demonstrated the occurrence of distinct isolate-specific switching repertoires in clinical isolates of *C*. *tropicalis*^13^. The switched strains exhibited altered *in vitro* virulence traits, including morphogenesis, biofilm formation and hemolysis capability, which could potentially affect their survival within the host^13^. Further, we showed that phenotypic switching in *C*. *tropicalis* is associated with changes in virulence. A switch strain named the crepe variant exhibited higher cytotoxicity to FaDu cells and higher virulence in a *Galleria mellonella* infection model compared to those of its parental counterpart strain (unswitched strain)^14^. Currently, the ways that switching affects the pathogen interactions with the host and the role of switching in the pathogenesis of *C*. *tropicalis* are largely unexplored.

Larvae of the greater wax moth *G*. *mellonella* are a well-accepted model for the study of fungal pathogen-host interactions and serve as complementary hosts to conventional vertebrate animal models^15,16^. This invertebrate model exhibits several traits that make it a useful model for fungal pathogenesis studies. *G*. *mellonella* larvae can be reared at 37 °C. This characteristic allows microorganisms to be studied under the temperature conditions at which they are pathogenic to human hosts^17^. An additional advantage of *G*. *mellonella* is that its innate response is evolutionarily conserved in relation to mammals and possesses both cellular and humoral defenses^15,18^. The cellular immune response is mediated by phagocytic cells, termed hemocytes^19^, while the humoral defenses involve the production of effector molecules such as melanin and antimicrobial peptides^20^.

**Figure 1:**
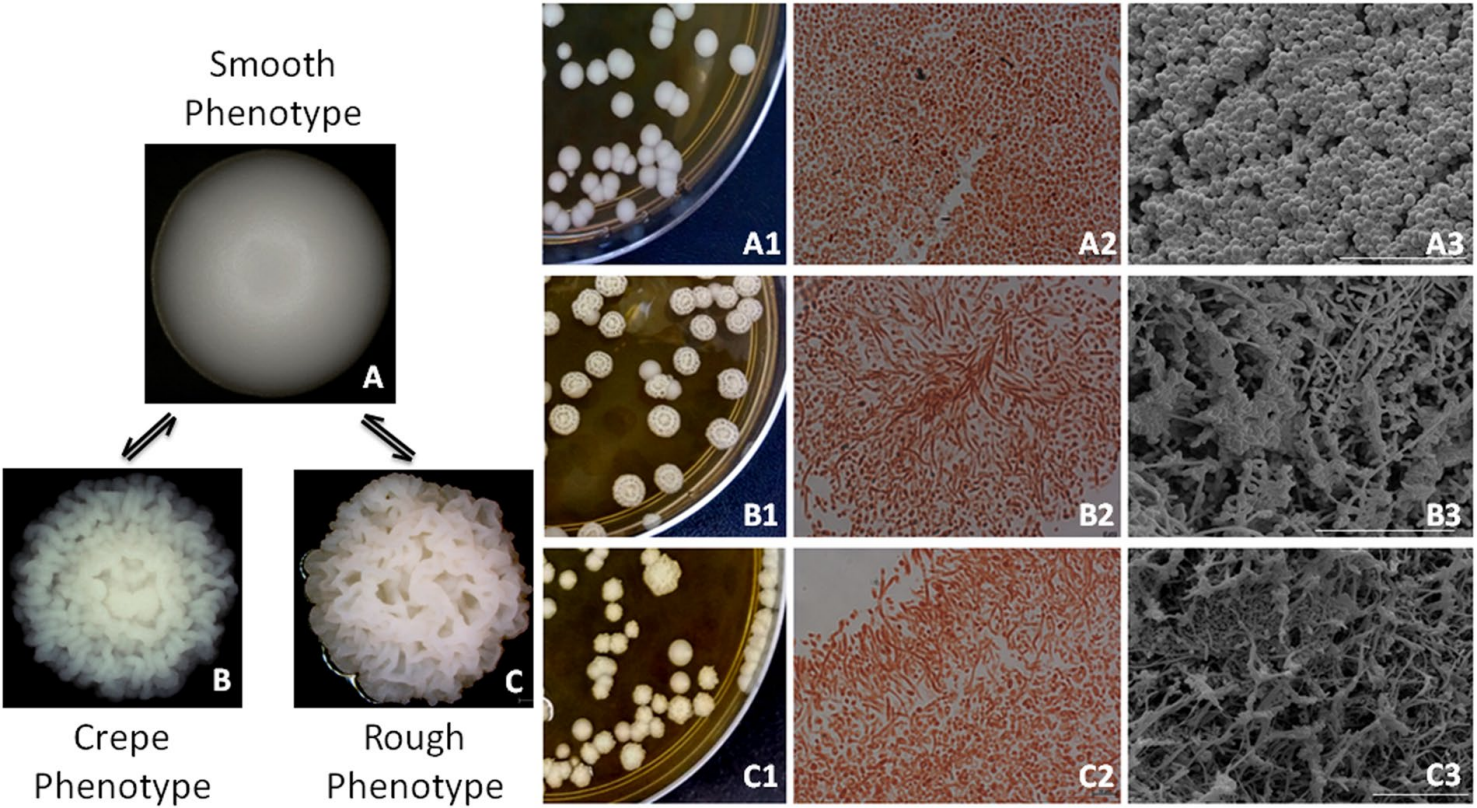
Representative colonies of morphotypes of the phenotypic system 49.07 of *C*. *tropicalis*. (**A**) Smooth phenotype (parental strain and revertant of the crepe and rough types); (**B**) crepe phenotype (variant crepe); (**C**) rough phenotype (variant rough). Top line shows images of parental strain – clinical strain from tracheal secretion. Middle line shows images of crepe variant. Bottom line shows images of rough variant. Colonies phenotypes following 96 h incubation on YPD agar at 28 °C: (A1) parental strain; (B1) crepe variant and (C1) rough variant were photographed using a stereoscopic microscope (Nikon SMZ-745). Light microscopy of safranin-O cell staining of cells types in the colonies (blastoconidia and filamentous forms) (63×): (A2) parental smooth colony; (B2) crepe variant colony and (C2) rough variant colony. Scanning electron micrographs of cells types in the colonies: cells from parental smooth colony (A3); cells from crepe variant colony (B3) and cells from rough variant colony (C3). Scale bars = 50 µm.

As the clinical isolates of *C*. *tropicalis* undertake phenotypic switching at high frequencies^12,13^ and because this event affects the virulence of *C*. *tropicalis*^14^, in the present study, we sought to evaluate whether the phenotypic switching may alter host-pathogen interactions using a *G*. *mellonella* model. To this end, we employed a switching system that was previously described by our group^14^ that comprises five colony phenotypes (morphotypes): the original phenotype (smooth colony dome); the two switch variants, exhibiting an irregular and structured dome surface (crepe and rough); and the switching revertants of the crepe and rough types (strains that switched back from the variants to the original phenotype), as illustrated in Fig. 1. The colonies of the strains presenting a smooth phenotype (original and revertant strains) consist entirely of budding yeast cells; in contrast, the cell morphologies in the colonies of the switched variants consist of budding yeast cells and of the filamentous forms (Fig. 1). In the present study, we evaluated several traits, including the melanization, expression of galiomicin and gallerymicin-encoding genes, phagocytosis, hemocyte density and the capacity of hemocytes to kill *Candida* cells. These findings could provide information on the insect immune response to the morphotypes of *C*. *tropicalis* and the role of switching on *C*. *tropicalis* pathogenesis.

## Results

### Switched strains of *C*. *tropicalis* promote increased melanization in *Galleria mellonella*

Previous work has established the suitability of the larvae of *G*. *mellonella* for detecting variations in the virulence of the switched strains of *C*. *tropicalis*, where a variant strain named crepe was more virulent than its original counterpart (parental strain), in contrast to a variant strain named rough that was less virulent than the parental strain^14^. In the present work, we aimed to evaluate the influence of phenotypic switching on larval humoral defense, particularly the production of melanin, which is a key step in the antimicrobial response of *G*. *mellonella*. We examined larvae melanization postinfection with the variants (crepe and rough) and their revertant (CR and RR) strains in comparison to the melanization of larvae that were infected with the parental strain (clinical isolate). This parameter can be monitored by measuring the OD at 405 nm of the hemolymph obtained from infected larvae.

**Figure 2:**
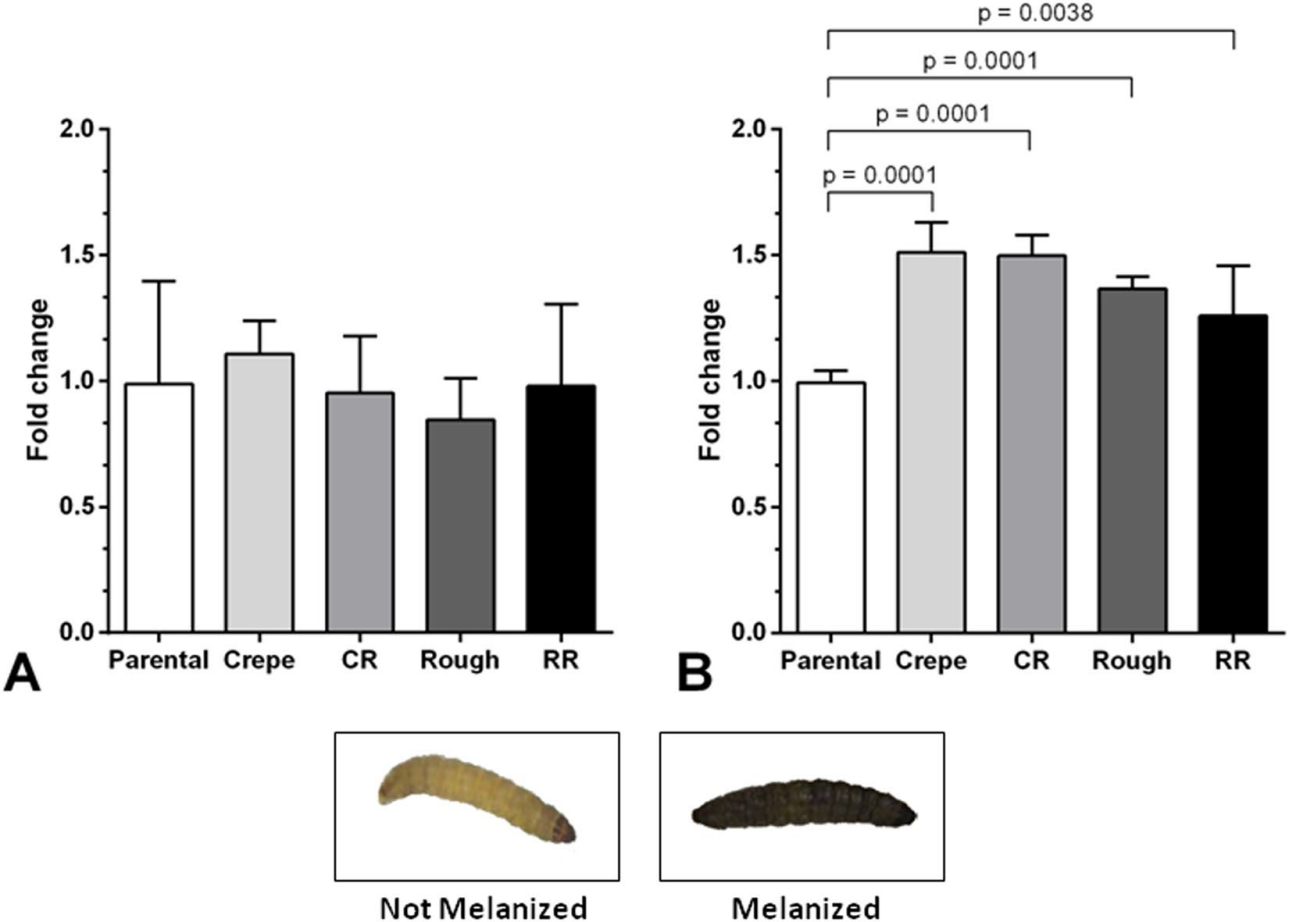
Melanization in *Galleria mellonella* larvae post-infection 2 h (**A**) and 24 h (**B**) with 5 × 10^5^ cell per larvae of morphotypes of the 49.07 phenotypic switching system of *Candida tropicalis* (parental strain, crepe variant, rough variant, revertant of crepe [CR] and revertant of rough [RR]). Melanization of the switched morphotypes relative to the melanization of the parental morphotype. Not melanized: larvae injected with PBS (control). Melanized: a typical dark color due to accumulation of melanin after larvae infection.

In all of the larvae that were infected with the different strains, a typical dark color was observed due to the accumulation of melanin, in contrast with the nonmelanized larvae that had been injected with PBS (control), as illustrated in Fig. 2. Melanization was observed in the hemolymph of larvae that had been infected with all strains of the switching system (parental, switched strains and revertants). At 2-h postinfection, the larvae that had been infected with the switched strains showed the same extent of melanin production as that observed for the larvae that had been infected with the parental strain (Fig. 2A). At 24-h postinfection, both variants (crepe and rough) induced higher melanization in *G*. *mellonella* larvae than that in their parental counterpart (original strain) (*p* = 0.0001). Further, the revertant of crepe (CR) and the revertant of rough (RR) also induced higher melanization than the parental strain (Fig. 2B), despite their parental-like colony morphology. These data suggest that phenotypic switching in *C*. *tropicalis* generates strains that are differently recognized by *G*. *mellonella* larvae.

### Phenotypic switching in *C*. *tropicalis* causes the altered expression of genes encoding antimicrobial peptides by *G*. *mellonella larvae*

Antimicrobial peptides, or host-defense peptides, play a major role in innate immunity, showing broad-spectrum microbicidal activity. We analyzed the expression of the galiomicin and gallerymicin peptide-encoding genes, which are associated with the immune response of *G*. *mellonella*, to evaluate the insect humoral response postinfection with the switched strains of *C*. *tropicalis*. Larvae were infected with the unswitched (parental) and switched strains, and RNA was extracted at 4-h postinfection. Transcriptional activation is represented by the fold change of the expression of the galiomicin and gallerymicin genes in the parental strain-infected *G*. *mellonella* and the switched strains-infected *G*. *mellonella* relative to that in the PBS-injected control larvae and was normalized using the housekeeping gene β-actin.

**Figure 3:**
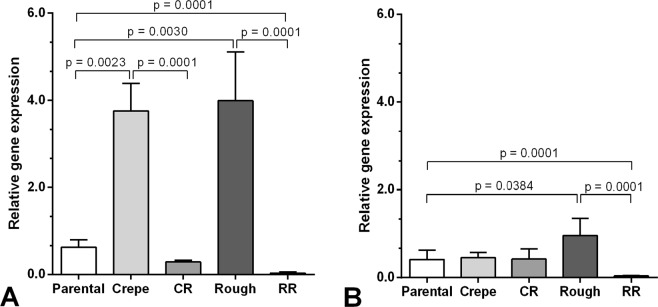
Relative gene expression of peptides galiomicin (**A**) and gallerymicin (**B**) 4-h postinfection with 5 × 10^5^ cell per larvae of all morphotypes of *Candida tropicalis* (parental strain, crepe variant, rough variant, revertant of crepe [CR] and revertant of rough [RR]). Data were normalized with the β-actin gene and relativized with the expression of larvae inoculated with PBS solution.

Analysis by real-time quantitative PCR showed that the levels of galiomicin were higher in insects infected with the crepe variant (p = 0.0023) and rough variant (p = 0.0030) than those in insects infected with the parental strain (Fig. 3A). Interestingly, both revertants (CR and RR) exhibited lower galiomicin expression than that in their variant counterparts, where the revertant of crepe exhibited galiomicin expression levels that were restored to the levels that were observed in response to the parental strain.

The amount of gallerymicin mRNA at 4-h postinfection did not differ between the insects infected with the crepe variant and those infected with the parental strain. Only larvae infected with the rough variant showed a higher expression level of gallerymicin compared to that in the larvae infected with the parental strain (p = 0.0384) (Fig. 3B). The RR exhibited lower gallerymicin expression compared to its variant counterpart and its parental strains.

### Switched strains are phagocytosed by *G*. *mellonella* hemocytes at distinct rates

The fluctuation in the phagocytosis rate has been related to the ability of microorganisms to kill larvae. We examined whether cells of the switched strains differ in the extent to which they are phagocytosed by *G*. *mellonella* hemocytes compared to that observed for the parental strain.

**Figure 4:**
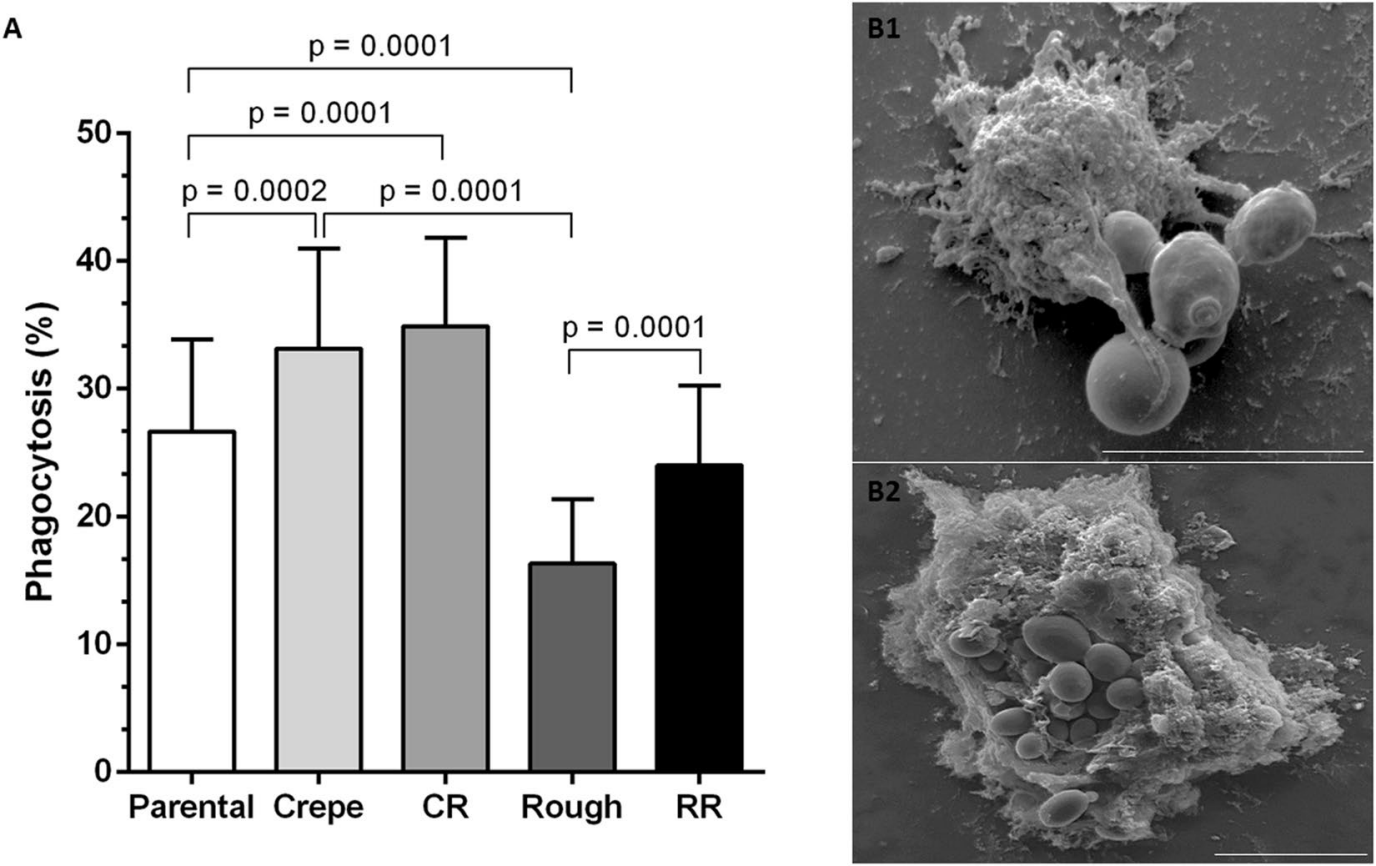
Percentage of phagocytosis of *Candida tropicalis* morphotypes by *Galleria mellonella* hemocytes co-cultured in a ratio of 5:1 (*Candida* morphotypes cells/hemocyte cells) for 2 h (A). Morphotypes: parental strain, crepe variant, revertant of crepe (CR), rough variant and revertant of rough (RR). Scanning electron microscopy photomicrographs showing interactions of *C*. *tropicalis* parental strain with *G*. *mellonella* hemocytes after 2 h of infection (B1, B2). Scale bars = 10 µm.

All morphotypes (parental, switched strains and revertants) were phagocytosed by *G*. *mellonella* hemocytes after 2 h of *Candida* cell/hemocyte cocultivation (Fig. 4A). The crepe variant was more effectively phagocytosed by *G*. *mellonella* hemocytes than the parental strain (p = 0.0002), whereas the rough variant was less phagocytosed (p = 0.0001) than the parental strain, indicating that the strains are differently recognized by the immune components. The amount of phagocytosis of the crepe revertant was also significantly higher (p = 0.0002) than the amount of phagocytosis observed with the parental strain, while the revertant of rough showed the same extent of phagocytosis as the parental strain (Fig. 4A), and thus, the reversion restored the phagocytic susceptibility of the original clinical strain.

The electron micrograph shows the *G*. *mellonella* hemocyte-*Candida* cell interactions (Fig. 4B1, B2). Hemocytes were able to engulf the cells of all of the strains of the switching system (data not shown). The *Candida* cells were surrounded by hemocyte membrane projections (Fig. 4B1) and were internalized by hemocyte cells (Fig. 4B2).

### Infection with switched *C*. *tropicalis* strains leads to alterations in the density of circulating hemocytes

**Figure 5:**
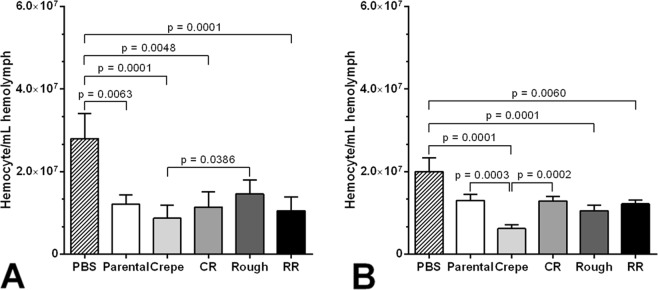
Hemocyte density post-infection with morphotypes of *Candida tropicalis* (5 × 10^5^ cells per larvae) for 4 h (**A**) and 24 h (**B**). Morphotypes: parental strain, crepe variant, revertant of crepe (CR), rough variant and revertant of rough (RR). PBS (control).

Hemocytes are important to the larva’s cellular defense against fungi since they act as phagocytic cells. Thus, we aimed to examine whether the switched strains had any effect on the hemocyte density. The majority of the strains induced a decrease in the hemocytes present in the hemolymph of *G*. *mellonella* compared with that of the control (PBS) (Fig. 5). At 4-hours postinfection, no differences in the hemocyte density were observed when the larvae were infected with the switched strains (variants and revertants) compared to that observed for infections with the parental strain (Fig. 5A). Differences in the hemocyte density were observed only between larvae infected with the rough and crepe variant strains. At a longer infection period (24 h), the hemocyte population was significantly lower in the larvae infected with the crepe variant strain than in those infected with the parental strain (p = 0.0003). For the larvae infected with all the remaining switched strains (crepe revertant, rough variant and rough revertant), no differences in the hemocyte density were observed compared to that in the parental strain (Fig. 5B). Interestingly, the larvae infected with the CR showed higher hemocyte density (p = 0.0002) compared to the larvae infected with its variant counterpart, restoring the levels to those observed for larvae infected with the parental strain (Fig. 5B).

### Capability to survive in the presence of *G*. *mellonella* hemocytes *in vitro* varies among the switched phenotypes

**Figure 6:**
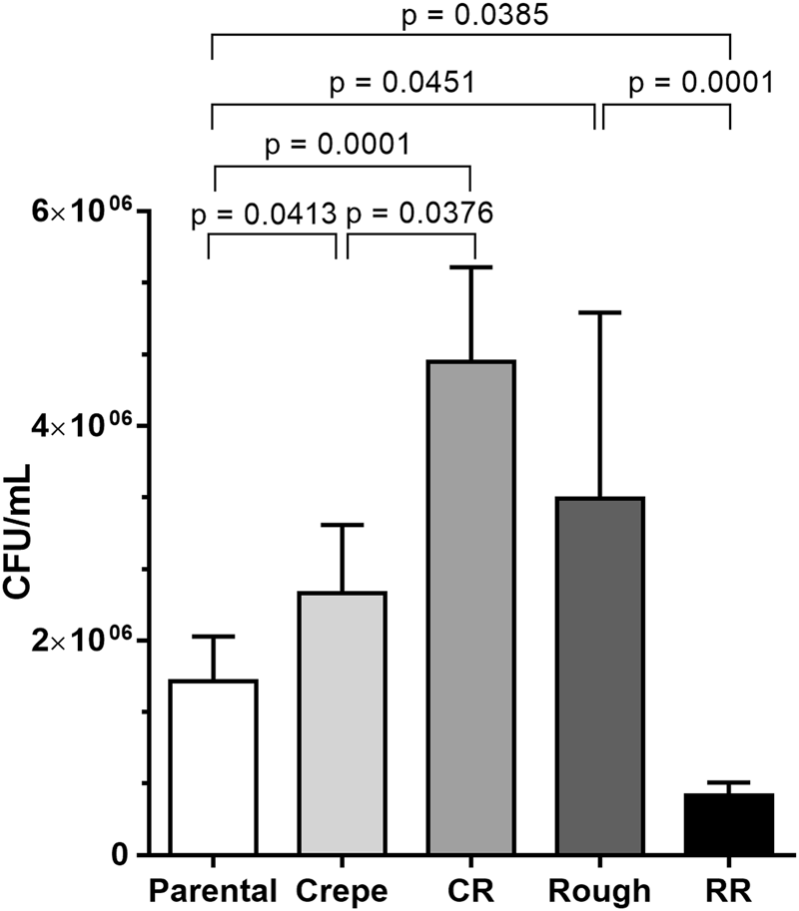
Colony forming units (CFU) counts 4 h after *in vitro* co-incubation of hemocytes (1 × 10^5^) and *C*. *tropicalis* morphotypes cells (5 × 10^5^ cells). Morphotypes: parental strain, crepe variant, revertant of crepe (CR), rough variant and revertant of rough (RR). Candidacidal activity was determined by plating yeast cell suspensions onto YPD agar plates. Plates were incubated for 96 h and the resulting number of colonies enumerated (CFU/mL).

To determine the candidacidal activity of hemocytes against the distinct morphotypes, hemocytes were extracted from larvae and coincubated *in vitro* with the morphotype cells. Killing was determined by colony forming unit (CFU) counts 4-h after coincubation. As shown in Fig. 6, the cell viability of the crepe and rough switched variants was higher than the cell viability of their parental counterpart strain. The cell viability of the crepe revertant was also significantly higher (p = 0.0001) than that of the parental strain, while the revertant of rough strain showed a significantly lower viability than that of the parental strain (p = 0.0385) (Fig. 6). This result indicates that larval immune cells possess candidacidal activity and that the resistance of the switched strains to *G*. *mellonella* hemocytes is variable.

## Discussion

*Candida* species are common human fungal pathogens that cause a wide range of clinical diseases. The increase in the isolation of *C*. *tropicalis* in cases of both superficial and systemic infections worldwide makes it one of the most important *Candida* species. In some settings, bloodstream infections due to *C*. *tropicalis* have been associated with higher mortality than infections with other *Candida* species^21,22^.

The *G*. *mellonella* infection model has recently been shown to be a suitable model for analyzing fungal virulence factors. For instance, differences in virulence between the phenotypic states of *C*. *tropicalis* were reported using this infection model^14^. Since differences in virulence may be due, at least in part, to differential interactions with the host immune components, we examined some of the humoral and cellular immune responses of *G*. *mellonella* that were challenged with the morphotypes of the switching system 49.07 (Fig. 1). An understanding of these interactions is important for proposing new strategies to eliminate invasive pathogens and to prevent host death.

During infection experiments, colonies phenotypes of recovered cells from *G*. *mellonella* larvae were checked and their frequencies verified by CFU counting. Cells of each morphotype (parental and switched strains) exhibited their original colony phenotype at same rates to that previously described for the 49.07 system where on average, reversibility occurred at a frequency of 1%^13,14^. Considering that *G*. *mellonella* humoral immune response comprises melanin, which plays a crucial role in defense reactions^23^, we evaluated the production of melanin by *G*. *mellonella* larvae that were challenged with the distinct morphotypes. The high melanin production observed in larvae infected with the switched phenotypes (Fig. 2) indicates that phenotypic switching generates strains that alter the larval humoral response. The increase of melanization in response to the switched strains may be due to the differential activation of the enzyme phenoloxidase, which is involved in the pathway leading to the formation of melanin^24^. Melanization reaction and the prophenoloxidase (inactive form of phenoloxidase) system are closely related in *G*. *mellonella*^25^. This activating system is one of the main immune responses in invertebrates where recognition of cell surface molecules by pattern recognition proteins triggers the prophenoloxidase activation cascade. The activated phenoloxidase oxidizes phenols to quinones, which subsequently polymerize non-enzymatically to melanin^26^. It has been recently showed that *G*. *mellonella* larvae infected with *C*. *albicans*, a closely related species to *C*. *tropicalis*, exhibited a significant increase in the expression of prophenoloxidase and phenoloxidase-activating proteinase encoding genes^27^. Considering that the phenoloxidase cascade and resulting melanization of *G*. *mellonella* larvae occurs during *Candida* infections and that this response has been associated with cell wall β-glucans^28^, the increased melanization of larvae challenged with switched strains of the *C*. *tropicalis* 49.07 system suggests that switch states may exhibit altered arrangements of the cell wall components.

We further evaluated the alterations in the humoral immune response by examining the expression of antifungal peptide-encoding genes. Using RT-PCR, we evaluated the change in the expression of the gene encoding galiomicin, a defensin identified in *G*. *mellonella*, and gallerymicin, a cysteine-rich defensin-like antifungal peptide. Our experiments demonstrate that both genes were highly expressed in larvae infected with the rough variant compared to the expression observed in larvae infected with the unswitched parental strain, and the difference was more pronounced for the galiomicin-encoding gene than that for the gallerymicin-encoding gene (Fig. 3A). This is consistent with the reduced capacity of the rough variant to kill *G*. *mellonella* larvae compared to that of the parental strain^14^. It has been demonstrated that thermal or physical stress can lead to the increased expression of galiomicin, and that this can be associated with a decreased susceptibility of *G*. *mellonella* larvae to infection by *C*. *albicans*^29,30^. In contrast, the crepe variant that exhibited the highest virulence against *G*. *mellonella* larvae among all of the morphotypes of the 49.07 system, including the parental strain^14^, also induced higher expression of galiomicin than that of the parental strain. Altogether, the data suggest that the switched variants of *C*. *tropicalis* can transiently induce the expression of antifungal peptides and that the role of galiomicin during infection with distinct switched strains may be variable, demonstrating that pathogenicity is a multivariable and complex process. To the best of our knowledge, this is the first report in the literature that analyzes the antimicrobial peptide expression in *G*. *mellonella* that were challenged with *C*. *tropicalis* morphotypes that arose from a switching event.

A phagocytosis assay showed that hemocytes phagocytosed *C*. *tropicalis* cells after 2 h of *in vitro* coincubation. Further, our data revealed that the switched strains interact differentially with hemocytes (Fig. 4). The cells of the crepe variant and its switched revertant strain (CR) were preferentially phagocytosed by *G*. *mellonella* hemocytes compared to the cells of the parental strain; these data did not correlate with the effectiveness of killing the pathogen since the crepe variant was more virulent to *G*. *mellonella* larvae than the parental strain, as described previously^14^. For *Candida* species, hyphae and pseudohyphae contribute to virulence, promoting the evasion of phagocytosis. Mesa-Arango *et al*.^31^ described the presence of the pseudohyphae of *C*. *tropicalis* inside *G*. *mellonella* hemocytes. We demonstrated the capability of the crepe variant to develop *in vitro* filamentous forms to a greater extent than that of the parental strain^13^. Pseudohyphae formation could lead to the death of the hemocyte population. In contrast, the rough variant cells were less efficiently phagocytosed by hemocytes than the parental cells and the cells of the crepe variant, suggesting that the switching event in *C*. *tropicalis* may generate strains with a better escape mechanism from phagocytosis. Differences in phagocytosis among the distinct morphotypes (parental and switched strains) indicate that the strains are differently recognized by the *G*. *mellonella* immune components. Further, we showed that hemocytes can internalize a large number of *C*. *tropicalis* cells after 2 h of coincubation (Fig. 4B2). Studies are in progress to determine whether the switch state affects the composition or the rearrangement of specific components of the *C*. *tropicalis* cell wall, leading to altered recognition by hemocytes. As previous mentioned, it has been demonstrated that β-glucans in the yeast cell wall can activate some of the *G*. *mellonella* immune responses^28,32^.

Changes in the hemocyte density are another important factor to be examined in the immune response of *G*. *mellonella* larvae when studying fungal virulence^33^. A reduction in the number of hemocytes in the hemolymph has been reported in *G*. *mellonella* after infection with *C*. *tropicalis*^31^, so to further examine the properties of the switched strains that may impact host-pathogen interactions, we compared the hemocyte density of the larvae infected with the switched strains and parental strain. We observed the lowest numbers of hemocytes after 24 h of infection with the crepe variant among those associated with infections with the parental strain and with the other switched strains, possibly providing an explanation for the shorter survival with the crepe variant strain, as described previously^14^. A relationship between the ability of highly pathogenic yeast isolates to kill *G*. *mellonella* larvae and a reduction in the hemocyte density has been described^34^. We subsequently showed that the crepe variant and its revertant were more resistant to hemocytes than the parental strain, as revealed by CFU counting postincubation with hemocytes *in vitro* (Fig. 6). These data are in accordance with an *in vivo* assay in which larvae infected with these two strains exhibited a higher fungal burden than that observed for infections with the parental strain^14^. The rough variant was also more resistant to hemocytes than the parental strain, unlike its revertant strain, which exhibited the lowest resistance to hemocytes, suggesting a variable candidacidal ability for targeting the switched cells. Overall, the highly virulent crepe variant exhibited decreased hemocyte counts and increased resistance to hemocyte candidacidal activity compared to those of its parental strain counterpart. Interestingly, the CR strain, which arose by reverting from the crepe variant and thus exhibited smooth parental colony morphology, showed the same levels of hemocyte counts and virulence against *G*. *mellonella*, as well as the ability to induce galiomicin expression, as the parental strain (clinical isolate), revealing that some attributes of the host response, but not all, are restored following the switching reversion event in the pathogen. It is currently unknown the molecular mechanisms mediating phenotypic switching in *C*. *tropicalis* 49.07. Considering that transcriptional regulators form a complex regulatory network in *Candida* switching^35–37^, it is tempting to imagine that although parental and revertant strains of the 49.07 system show similar colony morphology, they exhibit distinct gene expression profiles. The mechanism responsible for the differences in host-pathogen interactions observed for the different colony types is currently not understood. A characterization of the cell wall of switched morphotypes could provide insights into the differential interactions with the host immune components observed for the variants and revertants strains in *C*. *tropicalis*. Currently, we still have much to learn about the role of switching in the pathogenesis of *C*. *tropicalis*.

In conclusion, in the present study, we found that phenotypic switching affected *C*. *tropicalis* virulence, at least in part, by altering the host-pathogen interactions in the *G*. *mellonella* infection model. The results indicate that phenotypic switching in *C*. *tropicalis* may generate strains that are differently recognized by the *G*. *mellonella* immune components, as well as generating strains that are more resistant to hemocyte candidacidal activity. These data may have the potential to further our understanding of *C*. *tropicalis* pathogenesis.

## Methods

### Microbial strains and culture conditions

Morphotypes (original phenotype-parental, crepe variant, crepe revertant, rough variant and rough revertant) of the switching phenotypic system 49.07 of *C*. *tropicalis* used in this study were stored as frozen stocks with 20% glycerol at −80 °C and subcultured on YPD agar plates (1% yeast extract, 2% peptone, and 2% dextrose) at 28 °C. Morphotypes were routinely grown in YPD liquid medium at 28 °C in a shaking incubator and plated on YPD agar plates at 28 °C for 96 hours.

### Fungal inocula preparation

Morphotypes were grown on YPD agar at 28 °C for 96 h. Colony cells were collected and resuspended in PBS. Yeast cells were counted using a hemocytometer, and the cell density was adjusted to 5 × 10^7^ cells/ml.

### *Galleria mellonella* rearing and larvae manipulation

*G*. *mellonella* larvae in the final stage without color alterations and with adequate weights (240–300 mg) were selected. The insect proleg regions were cleaned with 70% ethanol before the experiments. Each infection group and control group contained 10 randomly chosen larvae of the appropriate weight. Using a syringe (Hamilton, USA), 10 µl of inoculums with all morphotypes was injected into the hemocell through the last left proleg of the larvae, and the control group received only a PBS inoculum. The insects were maintained at 37 °C for 1, 2, 4 and 24 h under protection from light for further analysis. Each experiment was repeated at least 3 times.

### Hemolymph collection

The *in vitro* experiments required hemolymph collection. To collect the hemolymph, the larvae were selected according to the following criteria: last instar, weighing 240–300 mg, and presenting with clear, uniform color without dark spots or grayish marks. To collect the hemolymph, we held the larva in its ventral position, punctured one of the central prolegs and collected the hemolymph. Adipose tissue and any liquid of a dark color were discarded. The collected hemolymph of ten larvae was transferred to a microtube containing 900 μL of IPS (insect physiological saline: 150 mM sodium chloride (Promega, USA), 5 mM potassium chloride (Promega), 10 mM Tris HCl (Promega) pH 6.9, 10 mM EDTA (Promega) and 30 mM sodium citrate (Sigma-Aldrich, USA) plus 10 mM N-ethylmaleimide (Sigma-Aldrich) (anticoagulant). Tubes were placed on ice, and sample collection was carried out immediately to avoid cell melanization. After centrifugation at 2000 rpm and at 4 °C for 5 min, the supernatant was discarded, cells were washed with 500 μl of cold IPS and the contents of two tubes (or several, depending on the number of assays) were pooled together in a new tube. A second centrifugation was performed under the same conditions. The supernatant was again discarded, and the cells were resuspended in 1000 μl IPS. The number of cells was determined with a Neubauer chamber.

### Quantification of melanization

Melanization assays were performed according to Maurer *et al*.^38^, with modifications. Ten larvae per group were infected with the phenotypic strains and incubated at 37 °C. Hemolymph was collected at 2-h and 24-h postinfection and diluted 1:10 in IPS buffer supplemented with N-ethylmaleimide to prevent further melanization. A total of 100 µl of each sample was placed in 96-well microdilution plates, and an optical density (405 nm) reading was performed to quantify the melanization. The optical density values of hemolymph from the infected larvae were normalized to that of the control larvae (PBS inoculum). All experiments were performed in triplicate, as described above.

### RNA extraction and gene expression analysis (RT-qPCR)

Larvae were inoculated as described above. Three replicate samples were collected at 4-h postinfection, and each of the triplicate samples comprised at least three whole larvae. Larvae from each experimental group were snap-frozen in liquid nitrogen and ground to a powder by mortar and pestle in TRIzol (Invitrogen, United Kingdom). The samples were further homogenized, and RNA was extracted and purified using a RNA Mini Kit (Thermo Fisher Scientific, USA) according to the manufacturer’s instructions. RNA was quantified and the quality assessed using a NanoDrop spectrophotometer (ThermoScientific, Loughborough, United Kingdom). cDNA was synthesized from 200 ng of extracted RNA using an RT-PCR kit (Invitrogen, Carlsbad, CA, EUA) in a GeneAmp® PCR (Eppendorf, Gradiente Mastercycler) following the manufacturer’s instructions. Primers used for quantitative PCR (qPCR) were as follows: galiomicin forward, 5′-CCTCTGATTGCAATGCTGAGTG-3′; galiomicin reverse, 5′-GCTGCCAAGTTAGTCAACAGG-3′; gallerymicin forward, 5′-GAAGATCGCTTTCATAGTCGC-3′; gallerymicin reverse, 5′-TACTCCTGCAGTTAGCAATGC-3′; β-actin forward, 5′-GGGACGATATGGAGAAGATCTG-3′; and β-actin reverse, 5′-CACGCTCTGTGAGGATCTTC-3′, as described elsewhere^39^.

The cycling conditions consisted of 2 min at 50 °C, 10 min at 95 °C, and 40 cycles of 15 s at 51 °C and 60 s at 60 °C. Each sample was analyzed in duplicate using a StepOnePlus™ Real-Time PCR System (Applied Biosystems®). The reactions were performed using Platinum® SYBR® Green qPCR Supermix-UDG (Invitrogen, Carlsbad, CA, USA) with a final volume of 20 μl. Relative gene expression was calculated using the 2^−(Δ-ΔCt)^ method.

### Determination of hemocyte density

*G*. *mellonella* larvae (n = 10) were infected and treated as described above. After the infection period (4 and 24 h), hemolymph samples from 3 larvae of each group were collected in IPS supplemented with 10 mM of N-ethylmaleimide to prevent melanization and coagulation. The hemolymph was diluted 1:10, and the hemocyte density was estimated by using a hemocytometer under a light microscope. As a control, the samples were compared with those from PBS-injected larvae, and the results are expressed as a number of hemocytes/ml of hemolymph. Experiments were repeated at least 4 times.

### Phagocytosis assay

To study the phenotypic switching effect on the phagocytic capacity of the hemocytes, 13-mm round coverslips that were treated with acetic acid were placed in a 24-well plate. Each well was filled with a volume corresponding to 1 × 10^5^ hemocytes and was brought to a total volume of 1000 μl with RPMI 1640 medium (Gibco, USA) plus 10% fetal bovine serum (FBS, Gibco®, Brazil). The plates were incubated for 1 h at 37 °C with 5% CO_2_ to allow cell adherence. Then, nonadherent hemocytes were removed by washing with RPMI 1640 medium at room temperature, and the adhered cells were coincubated with each morphotypic strain at a 1:5 ratio. Phagocytosis was allowed to occur for 2 h; samples were then washed to remove the nonadherent yeast. In sequence, samples were fixed with 1 ml cold methanol (Sigma-Aldrich, USA) for 20 min. Then, cells were stained with May-Grünwald (Laborclin, Brazil) for 15 min, washed with Sorenson’s buffer (0.133 M Na_2_HPO_4_ and 0.133 M KH_2_PO_4_) and immersed in Giemsa dye (Laborclin, Brazil) for 15 min. Finally, the coverslips were washed again with Sorenson’s buffer, air dried, and mounted on glass slides. Under an optical microscope (1000 times magnification), cells from 20 fields were analyzed and quantified according to the number hemocytes containing internalized yeast cells.

### Phagocytic assay for microscopic analysis

A phagocytic assay for scanning electron microscopy (SEM) analysis was conducted as described by Tomiotto-Pellissier *et al*.^40^, with modifications. Round coverslips (13 mm) treated with poly-L-lysine (Sigma Aldrich, USA) were placed in a 24-well plate. Each well was filled with a volume corresponding to 5 × 10^5^ hemocytes and was adjusted to 1000 μl with RPMI 1640 medium plus 10% FBS. The plates were incubated for 1 h at 37 °C with 5% CO_2_ to allow cell adherence. Then, nonadherent hemocytes were removed by washing with RPMI 1640 medium at room temperature. Adhered cells were infected with each morphotypic strain at a ratio of 1:2. Phagocytosis was allowed to occur for 2 h at 37 °C with 5% CO_2_ in 1 ml RPMI 1640 medium plus 10% FBS. Next, each coverslip was washed with IPS and fixed by immersion in 1 ml of fixative [2% glutaraldehyde (Sigma Aldrich, USA) in 0.1 M sodium cacodylate buffer (Sigma Aldrich, USA), pH 7.2] for 24 h. After this incubation, the samples were postfixed for 1 h in 1% OsO_4_ (Electron Microscopy Sciences, Hatfield, PA) diluted in 0.1 M sodium cacodylate buffer, pH 7.2. The fixed material was dehydrated in an ethanol gradient (30, 40, 50, 70, 80, 90 and 100°GL), and the samples were dried to the critical point with CO_2_ (BALTEC CPD 030 Critical Point Dryer), while placed on an aluminum holder. Coverslips were covered with tape for partial cell surface removal, and then the samples were coated with gold (BALTEC SDC 050 Sputter Coater) and analyzed by SEM (model Quanta 200, FEI, Netherlands).

### Determination of the candidacidal activity of hemocytes

The capacity of hemocytes to kill *Candida* cells was determined with the colony forming unit (CFU) counts 4-h after coincubation. Hemocytes were extracted from larvae and incubated at a density of 1 × 10^5^ hemocytes in 1 ml RPMI 1640 medium at 37 °C. *C*. *tropicalis* morphotypes (5 × 10^5^ cells) were added, and killing was measured by diluting and plating yeast cell suspensions onto YPD agar plates. The plates were incubated for 96 h, and the resulting number of colonies was counted. The results were calculated from three experiments, with colony counts performed in triplicate for each sample.

### Statistical analysis

Three independent experiments were performed in triplicate for all experiments. All data were previously submitted to the F test and the Shapiro-Wilk test for analysis of variance and normality test, respectively. Comparison between the groups were done by one-way ANOVA, followed by Tukey’s test for multiple comparisons. P < 0.05 was considered statistically significant. All statistical analysis were performed by GraphPad Prism 5 statistical software (GraphPad Software, Inc., USA, 500.288). Data were expressed as mean ± standard deviation.
